# Liposomal mitoxantrone-based multidrug chemotherapy as a bridge to allogeneic hematopoietic stem cell transplantation in relapsed/refractory acute lymphoblastic leukemia (ALL) after immunotherapy failure: a case report

**DOI:** 10.3389/fmed.2024.1383288

**Published:** 2024-05-22

**Authors:** Jin Deng, Hongxia Chen, Yi Yang, Hua Ji, Hui Liu

**Affiliations:** ^1^Department of Hematology, Chongqing University Three Gorges Hospital, Chongqing, China; ^2^Department of Laboratory Medicine, Yancheng Third People’s Hospital, Yancheng, Jiangsu, China; ^3^Department of Bone Marrow Morphology, Chongqing University Three Gorges Hospital, Chongqing, China

**Keywords:** acute lymphoblastic leukemia, allotransplantation, immunotherapy, mitoxantrone liposomes, relapse/refractory

## Abstract

Acute lymphoblastic leukemia (ALL) represents a malignancy involving early-stage differentiated lymphoid cells that invade the bone marrow, blood, and extramedullary sites. First-line treatment spans 2–3 years with induction, consolidation, intensification, and long-term maintenance phases. Relapsed/refractory (R/R) ALL typically carries an adverse prognosis, and there is currently no standard of care for this disease. Here, we present a case of R/R ALL that responded effectively to liposomal mitoxantrone-based multidrug chemotherapy, resulting in a rapid complete response after 35 days of therapy. Subsequently, the patient was successfully treated with allo-HSCT. At 5 months follow-up, the patient was alive and leukemia-free. Additionally, no severe adverse events were recorded during liposomal mitoxantrone treatment or hospitalization for allo-HSCT. Given the encouraging efficacy and the manageable adverse events observed in our case, liposomal mitoxantrone-based multidrug chemotherapy should be further explored as a bridge to allo-HSCT in patients with R/R ALL.

## 1 Introduction

Acute lymphoblastic leukemia (ALL) refers to a malignant proliferation of lymphoid cells blocked at an early differentiation period with a poor prognosis (1) The prognosis for relapsed/refractory ALL (R/R ALL) is almost uniformly fatal, with 1-year survival rates of 26, 14, and 12% following the first, second, and third or later salvage attempts, respectively (2, 3). Therefore, there exists an urgent need to innovate and devise novel therapeutic strategies for managing R/R ALL.

Attaining an initial complete response (CR) followed by consolidative allogeneic hematopoietic stem cell transplantation (allo-HSCT) appears to present the most promising opportunity for achieving long-term remission. Nonetheless, the challenge persists for patients who failed to achieve CR before allo-HSCT, serving as a significant hurdle (4). Currently, blinatumomab has been indicated for the treatment of patients with R/R B-cell precursor ALL (5) and as a bridge therapy to allo-HSCT (6). In cases where patients do not achieve a CR after receiving multidrug chemotherapy (VDCP: vincristine, daunorubicin, cyclophosphamide, and prednisone) or immunotherapy with blinatumomab, selecting an appropriate treatment for those desiring a rapid response poses a challenge.

Mitoxantrone has demonstrated effectiveness in treating lymphoma, leukemia, and various solid tumor types (7). Liposomal mitoxantrone, the first approved mitoxantrone nano-drug, exhibits a favorable pharmacokinetic profile and significantly prolonged survival in preclinical studies compared to the equivalent dose of mitoxantrone (8, 9). Here, we report a patient diagnosed with R/R ALL who was successfully treated with liposomal mitoxantrone-based multidrug chemotherapy (VMP: vincristine, liposomal mitoxantrone, and prednisone) and used it as a bridge to allo-HSCT.

## 2 Case report

In November 2020, a 60-year-old woman was admitted to the Southwest Hospital of the Army Medical University for examination. A bone marrow (BM) smear showed hypercellular BM with 55.5% abnormal naive lymphocytes. Flow cytometric immunophenotyping indicated that the blasts were positive for HLA-DR, CD19, CD34, and CD38, while were negative for CD15, CD33, CD117, CD4, CD2, CD20, and CD56. Additionally, next-generation sequencing (NGS) of an ALL panel showed that no molecular genetic abnormalities were detected. Fluorescence *In Situ* Hybridization (FISH) testing for the ALL Ph chromosome, KMT2A rearrangement, and ETV6-RUNX1 fusion were also negative. Based on the above results, the patient was diagnosed with B-cell ALL (Ph-). The patient also had a history of type 2 diabetes and tuberculous pleurisy, with no family history of hematologic disorders.

Transferred to our institution, the patient underwent the first hyper CVAD-A chemotherapy, resulting in a CR as confirmed by bone marrow aspiration and an MRD of 0.23%. Subsequently, a total of 8 cycles of multidrug chemotherapy (Table 1) were administered for consolidation and intensification therapy. Maintenance therapy with mercaptopurine and methotrexate was received on 29 October 2021. From 15 November 2020 to 8 May 2022, the patient underwent lumbar punctures and received intrathecal injections with MTX/Ara/Dex eight times for central nervous system (CNS) prophylaxis. The results of each cerebrospinal fluid (CSF) examination showed a small number of cells approximately 1 million, and the flow cytometry test revealed no leukemic cells.

**Table 1:** Pre-relapse multidrug chemotherapy regimens.

Date	Chemotherapy regimens	Assessment of disease
11/17/2020	hyperCVAD/MA-A	CR, MRD+
12/15/2020	hyperCVAD/MA-B	CR, MRD+
1/17/2021	CAM	CR, MRD−
3/8/2021	HD-MTX + L-asp	CR, MRD−
4/4/2022	IA	CR, MRD−
5/12/2021	VDLD+PEG-asp	CR, MRD−
6/26/2021	COATD	CR, MRD−
8/3/2021	HD-MTX + PEG-asp	CR, MRD−
9/9/2021	EA	CR, MRD−
9/29/2021	6-MP + MTX (maintenance therapy)	
1/30/2022		CR, MRD−
5/9/2022	VICP	Recurrence
6/17/2022	Blinatumomab	Refractory, PR
7/6/2022	VP + Mit-Lip	CR, MRD+
8/30/2022	allo-HSCT (Haplo)	CR, MRD−

HyperCVAD/MA-A, cyclophosphamide + vindesine + doxorubicin + dexamethasone; hyperCVAD/MA-B, methotrexate + cytarabine; CAM, cyclophosphamide + cytarabine+6-mercaptopurine; HD-MTX, HD-methotrexate; L-asp., L-asparaginase; IA, idarubicin + cytarabine; VDLD, vindesine + doxorubicin + L-asparaginase + dexamethasone; HD-MTX, HD-methotrexate; PEG-asp., pegaspargase; COATD, cyclophosphamide + vindesine + cytarabine + etoposide + dexamethasone; EA, etoposide + cytarabine; VICP, vincristine + idarubicin + cyclophosphamide + prednisone; VP, vindesine + prednisone; Mit-Lip, mitoxantrone liposomes; allo-HSCT, CR, complete remission; MRD, minimal residual disease.

Following 6 months of maintenance therapy, the patient experienced bone marrow relapse. On 9 May 2022, a BM smear suggested that the predominant lymphocyte population was the abnormal naive lymphocyte (81%). Flow cytometric immunophenotyping showed that the blasts were positive for HLA-DR, CD19, CD10, CD38, and CD20dimc, but negative for TDT, CD34, CD33, CD58, and CD66c. Cytogenetic analysis revealed the following abnormalities: 45, XX (1;2) (p32; q37), add (10) (q32), −19, −20, +22. To achieve a rapid CR, the patient planned to undergo allo-HSCT following intensive chemotherapy. Hence, the patient received one cycle of VICP (vincristine, 4 mg qw; idarubicin, 10 mg d1–d3; cyclophosphamide, 1 g d1; and prednisone, 60 mg d1–d7) regimen as reinduction therapy. After 15 days of VICP therapy, the patient presented with abdominal pain, high fever, cough, and elevated inflammatory markers, which suggested the requirement to discontinue chemotherapy. Subsequently, the bone marrow was re-evaluated and showed that CR had still not been achieved. On 17 June 2022, the treatment was changed to receive one cycle of blinatumomab (9 μg d1–d3; 28 μg d4–d21). After 18 days of this treatment, there was still 79.5% abnormal lymphocytic leukemia in the BM, indicating that the patient had developed refractory disease.

Faced with this dilemma, on 6 July 2022, the patient’s treatment was switched to the VMP (vincristine 4 mg/d, d1, d8, d15, d22; liposomal mitoxantrone 10 mg d1–d3, d15–d17; prednisone 60 mg/d, d1–d7, reduce the dose by one third from day 8 to discontinuation) regimen. After 16 days (22 July 2022), the bone marrow examination revealed a significant reduction in the proliferation of nucleated cells, with approximately 23.0% protolytic lymphocytes and 3.90% abnormal naive B lymphocyte phenotypes. During the VMP therapy, the administration of liposomal mitoxantrone (10 mg) was delayed and resumed on 24 July 2022, due to the patient developing myelosuppression (grade 4 white blood cell and grade 3 platelet toxicity) and grade 2 lung infection, as per the National Cancer Institute Common Terminology Criteria for Adverse Events (NCI-CTCAE) version 4.03. In addition, no severe adverse events were reported. After 11 days (2 August 2022), the patient received the liposomal mitoxantrone (10 mg) as consolidation therapy; 8 days later, the examination results indicated that bone marrow morphology revealed 1% abnormal B lymphocytes, and the minimal residual disease (MRD) was 0.14%. Finally, the patient achieved a CR after 35 days of the liposomal mitoxantrone therapy.

On 12 August 2022, the patient received allo-HSCT at the second affiliated hospital of Chongqing Medical University. The donor was the patient’s daughter, with the HLA compatibility between donor and recipient of 7/12 in the GVH direction and 6/12 in the HVG direction. Donor stem cells were collected from peripheral blood mobilized by granulocyte colony-stimulating factor. After allo-HSCT, the patient was alive and leukemia-free at a 5-month follow-up.

Written informed consent was obtained from the patient for publication of this case report and any accompanying images.

## 3 Discussion

In the present study, we report a case with R/R ALL that achieved a CR after receiving liposomal mitoxantrone-based multidrug chemotherapy. Subsequently, the patient underwent successful allo-HSCT, which highlights the potential of liposomal mitoxantrone therapy in the treatment of R/R ALL and warrants further exploration.

Over the past decade, more promising and innovative immunotherapies and molecularly targeted drugs have been developed for leukemic cells while maintaining low toxicity to achieve MRD elimination and also drive better bridge hematopoietic stem cell transplantation (11). The evidence supports the use of blinatumomab as salvage therapy in both pediatric and adult patients with R/R B-ALL, even in cases involving molecular resistance (12). However, in cases where the disease persists and worsens despite receiving blinatumomab, how can patients derive any benefits from the treatment? Therefore, the challenges of achieving remission in these patients persist.

Paciucci et al. reported that the VMP (vincristine, mitoxantrone, and prednisone) regimen showed promising results in the treatment of ALL, with two patients with major refractory ALL achieving CR for over 28 and 31 months, respectively (13). It can be selectively concentrated in tumor tissues and steadily dispense the drug at an optimal rate within tumor microenvironments, thus demonstrating anti-tumor activity (13). It can be selectively enriched in tumor tissues and continuously released the drug at an appropriate rate within tumor microenvironments, thereby exerting anti-tumor activity (13). Simultaneously, liposome encapsulation reduced the damage to normal tissues and mitigated the cardiotoxicity related to anthracyclines. Additionally, by enhancing particle size and morphology, the common adverse events associated with liposome drugs, such as hand-and-foot syndrome and oral mucositis, were largely circumvented. Furthermore, liposomal mitoxantrone has shown encouraging efficacy and a favorable safety profile in peripheral T-cell lymphoma (8, 14).

In this study, the patient received the modified VMP (vincristine, liposomal mitoxantrone, and prednisone) therapy by replacing mitoxantrone with liposomal mitoxantrone. Encouragingly, the patient achieved a CR after 35 days of liposomal mitoxantrone treatment, with a manageable safety profile. In addition, the patient only developed myelosuppression (grade 4 white blood cell and grade 3 platelet toxicity) and grade 2 lung infection, without exhibiting common adverse events associated with mitoxantrone such as nausea and vomiting, stomatitis, and alopecia (15, 16). Liposomal mitoxantrone treatment also did not worsen the patient’s diabetes. Our case demonstrated that liposomal mitoxantrone could induce a rapid remission in ALL, offer patients a transplant, and prolong survival, suggesting that liposomal mitoxantrone might also be a promising treatment option for ALL in the future.

In conclusion, liposomal mitoxantrone is a promising drug, and liposomal mitoxantrone-based multidrug chemotherapy regimen is expected to be a bridge to allo-HSCT in patients with R/R ALL, especially those who urgently need to achieve a CR and undergo allo-HSCT after ineffective immunotherapy. Further studies with a large sample size are needed to confirm these findings in the future.

## Data availability statement

The original contributions presented in the study are included in the article/supplementary material, further inquiries can be directed to the corresponding author.

## Ethics statement

The studies involving humans were approved by the Ethics Committee of Chongqing University Three Gorges Hospital. The studies were conducted in accordance with the local legislation and institutional requirements. The participants provided their written informed consent to participate in this study. Written informed consent was obtained from the individual(s) for the publication of any potentially identifiable images or data included in this article.

## Author contributions

JD: Conceptualization, Data curation, Resources, Writing – original draft. HC: Conceptualization, Resources, Writing – original draft. YY: Conceptualization, Methodology, Project administration, Writing – review & editing. HJ: Formal analysis, Supervision, Writing – review & editing. HL: Investigation, Visualization, Writing – review & editing.
